# Accumulation of CD28^null^ Senescent T-Cells Is Associated with Poorer Outcomes in COVID19 Patients

**DOI:** 10.3390/biom11101425

**Published:** 2021-09-29

**Authors:** Mia J. Coleman, Kourtney M. Zimmerly, Xuexian O. Yang

**Affiliations:** 1 Department of Molecular Genetics and Microbiology, University of New Mexico School of Medicine, Albuquerque, NM 87131, USA; MJColeman@salud.unm.edu (M.J.C.); KMZimmerly@salud.unm.edu (K.M.Z.); 2 Class of 2023, University of New Mexico School of Medicine, Albuquerque, NM 87131, USA

**Keywords:** CD28^null^ T-cells, senescence, COVID-19, inflammation, cytotoxicity, immune decline

## Abstract

Coronavirus disease 2019 (COVID-19), a severe acute respiratory syndrome coronavirus 2 (SARS-CoV-2) causes infectious disease, and manifests in a wide range of symptoms from asymptomatic to severe illness and even death. Severity of infection is related to many risk factors, including aging and an array of underlying conditions, such as diabetes, hypertension, chronic obstructive pulmonary disease (COPD), and cancer. It remains poorly understood how these conditions influence the severity of COVID-19. Expansion of the CD28^null^ senescent T-cell populations, a common phenomenon in aging and several chronic inflammatory conditions, is associated with higher morbidity and mortality rates in COVID-19. Here, we summarize the potential mechanisms whereby CD28^null^ cells drive adverse outcomes in disease and predispose patients to devastating COVID-19, and discuss possible treatments for individuals with high counts of CD28^null^ senescent T-cells.

## 1. Introduction

SARS-CoV-2 infection (COVID-19) has a broad range of manifestations from asymptomatic carrier states to acute respiratory failure and death. COVID-19 also creates a surprising number of post-infectious complications, including transient hypercoagulability (predisposing patients to strokes and heart attacks), neurologic injury, and multisystem organ failure. Severity of infection is related to age and aging-associated, chronic inflammatory diseases such as diabetes, hypertension, cardiovascular disease (CVD), chronic obstructive pulmonary disease (COPD), and cancer. The molecular basis by which aging and the underlying conditions lead to severe COVID-19 remains poorly understood, although a growing body of studies demonstrates that hyper-reactive myeloid cells (monocyte and neutrophil), decreased CD8^+^ T-cell compartments, and severe lymphopenia contribute to COVID-19 severity [1,2,3,4]. Under-expression of IFN-I (and TLR7/TLR8) has been observed and discussed as a common characteristic between severe COVID-19 and the unfavorable conditions [5,6,7]. In this review, we focus on CD28^null^ (or CD28^−^) T-lymphocytes, another common feature shared by severe COVID-19, aging, and aging-associated chronic conditions, and discuss the potential mechanisms leading to poorer outcomes in COVID-19 and other infectious diseases.

CD28 is a costimulatory molecule expressed on the surface of all naïve T-cells. Under normal circumstances, a T-cell is activated via the T-cell receptor (TCR) interaction with a cognate antigen presented by the MHC complex and the costimulatory action of CD28 binding to a B7 molecule on the surface of antigen presenting cells (APCs) [8,9]. Failure of CD28–B7 costimulation during T-cell activation renders the cell anergic and unresponsive to antigenic stimulation.

**Figure 1:**
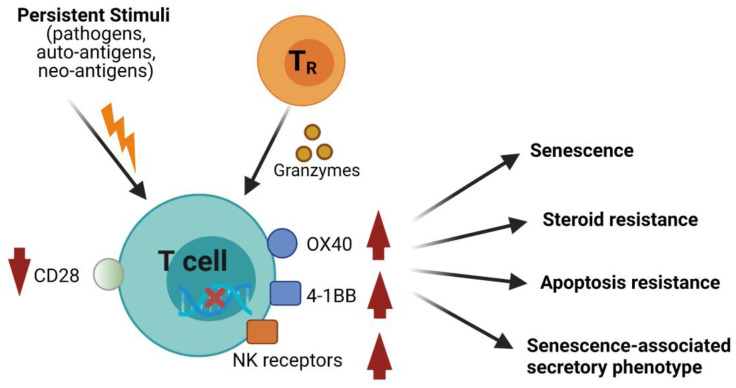
Molecular characteristics of CD28^null^ senescent T-cells. Persistent stimuli from various chronic conditions and/or interaction with regulatory T (T_R_) cells lead to a senescent phenotype of effector T-cells (see T_R_-mediated senescence in “Mechanisms underlying CD28^null^ cells-associated adverse consequences” below). These senescent T-cells down-regulate costimulatory molecule CD28 and express increased levels of surface molecules, OX40, 4-1BB and NK-like receptors. Because of accumulation of DNA damage and alteration of metabolic and epigenetic programs, these cells largely lose their proliferation ability. These cells are resistant to apoptosis and steroid treatment and gain a senescence-associated secretory phenotype (SASP).

Due to repeated antigenic stimulation during aging and chronic clinical conditions, T-cells lose their costimulatory molecule CD28 and become CD57-expressing effector senescent cells [10,11,12,13,14]. Senescence is a natural process of cells irreversibly losing the ability to replicate after a fixed number of replication cycles throughout their life. These cells have shorter telomeres and higher degree of DNA damage but are still metabolically active and capable of secreting inflammatory cytokines (Figure 1). It is important to note that CD28^null^ T-cells are not truly senescent; when a stimulation threshold is reached, they are able to proliferate [14,15]. Besides their senescent nature, both CD4^+^ and CD8^+^ CD28^null^ T-cells are resistant to apoptosis [13,16,17,18], which results in accumulation of these cells in aforementioned chronic conditions. CD28^null^ cells participate in several inappropriate immune responses that create a dually inflammatory and immunosuppressive state [14]. They have increased cytotoxic activity and compromise the development of other immune cells, leading to narrowed antigenic diversity and immune suppression (see detailed discussion below).

Expansion of the CD28^null^ populations is associated with several chronic inflammatory conditions including cancer, hypertension, CVD, diabetes, COPD, and chronic viral infection [10,12,19,20,21,22] (see more details in Table 1). Most recent studies demonstrate that COVID-19 patients with higher numbers of CD4^+^ CD28^null^, CD8^+^ CD28^null^, or CD4^+^ CD28^null^ and CD8^+^ CD28^null^ populations (or presented as lower numbers of CD28^+^ populations in some studies) have higher morbidity and mortality rates [23,24,25]. These results suggest that immunosenescence plays an important role in COVID-19. Interestingly, compared with healthy individuals, COVID-19 patients have higher numbers of CD57^+^ and/or PD-1^+^ (also CD28^null^) senescent/exhausted T-cells in both CD4^+^ and CD8^+^ compartments, suggesting that COVID-19 may also lead to the development of senescent/exhausted T-cells [26]. This phenomenon is associated with hyper-release of pro-inflammatory cytokines, IFNγ, IL-2, TNFα and IL-17 (IL-17A). Because of the association among the severity of COVID-19, the accumulation of CD28^null^ T-cells, and aging and aging-related underlying conditions, one may ask: Do CD28^null^ T-cells contribute to the worse outcome of COVID-19? Here, we analyze the pathogenic role of CD28^null^ senescent T-cells, outline their detrimental effects that may lead to severe COVID-19, and discuss potential treatments for individuals with high CD28^null^ counts.

## 2. Negative Consequences of CD28^null^ Cells in Aging and Underlying Conditions

### 2.1. Aging

Aging is accompanied by chronic inflammation; therefore, it is termed as “inflammaging” [66,67]. During aging, immunosenescence is an important process occurring in the immune system. In the T-cell compartment, chronic antigenic stimulation leads to accumulation of oligoclonal CD28^null^ T-cells (especially CD8^+^ CD28^null^ T-cells) in the elderly [10,11,12,14,68]. Excessive CD28^null^ T-cells occupy limited immunological spaces (“niches”), decreasing the development of new T and B cells [10,14,27,69]; subsequently, this results in low antigenic diversity and decreased immune responses to novel invasions [10,14,69]. Interestingly, CD8^+^ CD28^null^ cells in the elderly act as immune suppressors and contributes to faster progression of Alzheimer’s disease, an aging-associated disease [28]. Besides down-regulation of CD28, intensive replication of CD8^+^ T-cells causes the cells to express natural killer (NK) cell activating receptors, including CD94/NKG2 heterodimers and NKG2D/NKG2D homodimer [10,14]. With appropriate stimulation, CD8^+^ CD28^null^ cells produce increased amounts of IFNγ, which in turn up-regulates IL-15. IL-15 is an activator of CD28^null^ T-cells (and NK cells), and induces pro-inflammatory cytokine IL-6 [29,70].

Similar to CD8^+^ CD28^null^ cells, CD4^+^ CD28^null^ cells express activating NK cell receptors and are also inflammatory and cytotoxic [10,13,31]. Senescence of CD4^+^ CD28^null^ cells is associated with decreased levels of DNA methyltransferase 1 (Dnmt1) and Dnmt3a, causing overexpression of CX3CR1 and the cytotoxic markers KIR2DL4, perforin and CD70 [32,33]. Up-regulation of CX3CR1 promotes migration of the cells to inflammatory tissues, while hyper-expression of KIR2DL4, perforin, and CD70 heightens the cytotoxic and inflammatory effects. The pro-inflammatory feature of CD28^null^ T-cells, termed as senescence-associated secretory phenotype (SASP) [71], participates in the chronic inflammation observed in elderly and increases the risk of inflammaging associated CVD, chronic kidney disease, diabetes mellitus, cancer, depression, dementia, etc. [66,67].

In summary, expansion of CD28^null^ T-cells in aging individuals contributes to decline of protective immunity and elevation of pathogenic inflammation. Consequently, the decline of immunity in the elderly puts them at an increased risk of a serious illness when infected by SARS-CoV-2.

### 2.2. Diabetes

CD4^+^ CD28^null^ T-cell population is increased in both type 1 (T1D) and type 2 (T2D) diabetes [13,38,39]. In T1D and T2D patients, expansion of CD4^+^ CD28^null^ population is associated with poorer glycemic control and higher excretion of albumin [39], which predispose a risk of development of acute coronary syndrome [13]. Notably, in T2D patients who experienced a cardiovascular event, higher percentage of CD4^+^ CD28^null^ cells is associated with adverse outcomes [21]. Phoksawat et al. showed that patients with T2D have elevated numbers of CD4^+^ CD28^null^ NKG2D^+^ cells that release pro-inflammatory cytokine IL-17, contributing to systemic inflammation [38].

T1D is associated with lower numbers of CD8^+^ CD28^null^ cells, while patients who already have T2D or prediabetes have elevated levels of CD8^+^ CD28^null^ cells [34,35,36,37]. Due to the suppressive nature of CD8^+^ CD28^null^ cells, Yarde et al. attributed the overactive immune system in T1D to the lack of this cell population [34]. Unlike T1D, Lee et al. found CD8^+^ CD57^+^ and CD8^+^ CD28^null^ cell frequencies are significantly higher in prediabetes and T2D and proposed the frequency of senescent CD8^+^ T-cells as a predictive marker for development of hyperglycemia [36]. In line with this, Yi et al. revealed that increased numbers of CD8^+^ CD28^null^ cells alter the metabolic pathway and contribute to the development of T2D [37]. CD8^+^ CD28^null^ cells use glycolysis more than oxidative phosphorylation, producing higher amounts of reactive oxygen species (ROS) as a metabolic byproduct. ROS causes pro-inflammatory cytokine release and widespread inflammatory responses, contributing to the destruction of islet cells in the pancreas and the development of T2D [37]. Therefore, CD8^+^ CD28^null^ cells may contribute to the pathogenesis of hyperglycemia and T2D but not T1D.

Infection is a common cause of insulin resistance. The interaction of COVID-19 and diabetes causes severe insulin resistance and poor prognosis [72,73].

### 2.3. COPD

COPD is a chronic inflammatory lung disease associated with a history of exposure to cigarette smoke and other environmental pollutants. In addition to airway infiltration by neutrophils and macrophages, COPD patients have increased numbers of CD8^+^ T-cells in their lungs and peripheral blood, which contain higher counts of CD8^+^ CD28^null^ NKT-like cells [22,40,74]. Smoking increases the proportion of CD8^+^ CD28^null^ cells, and this proportion does not decrease when the patient quits smoking, suggesting that a self-perpetuating inflammatory feedback loop sustains this population of cells [40]. The CD8^+^ CD28^null^ cells are steroid resistant due to loss of glucocorticoid receptor (GCR), which makes clinical treatment difficult to achieve [40,41,74]. These cells produce heightened levels of cytotoxic mediators, perforin and granzyme B, and pro-inflammatory cytokines, IFNγ and TNFα. Their inflammatory phenotype is associated with a decrease in the expression of SIRT1, a class III NAD-dependent histone deacetylase (HDAC), which modulates the activity of transcription factors and reduces inflammation [42]. Accordingly, loss of CD28 in CD8^+^ CD45RA^+^ T-cells leads to a maturation-activation state, corresponding with a higher potential for tissue injury in COPD [43].

In addition to CD8^+^ CD28^null^ T-cells, two studies have shown that COPD patients have significantly higher numbers of CD4^+^ CD28^null^ populations in the lungs or blood [44,45], whereas another study found only a slight trend of increase in these cells [40]. Like CD8^+^ CD28^null^ cells, the CD4^+^ CD28^null^ cells express NKT-like receptors, CD94 and CD158 (KIR2DL1/S1/S3/S5), along with increased levels of perforin, granzyme B, and TNFα [44,45]. Lung infiltrating CD4^+^ cells (about 20% of which are CD28^null^ cells) from COPD patients exhibit a stable proliferative response when exposed to lung-specific elastin and collagen, implicating a possible autoimmune origin of the CD4^+^ CD28^null^ population [44].

In summary, accumulation of CD8^+^ and CD4^+^ CD28^null^ T-cells that produce cytotoxic and inflammatory mediators contributes to the tissue destruction and disease progression in COPD. Since COVID-19 primarily affects the respiratory system, COPD patients who contract SARS-CoV-2 are in danger of greater disease severity.

### 2.4. Hypertension

Recent studies linked errant adaptive immunity with hypertension. Oxidative stress in affected organs leads to the generation of neoantigens, including isolevuglandin-modified proteins, which are thought to elicit adaptive immune responses. Upon hypertensive stimuli, such as angiotensin II and high sodium levels, T-cells become pro-inflammatory and migrate to brain, blood vessel adventitia, periadventitial fat of heart, and kidney. T-cell-derived cytokines, such as IFNγ and TNFα (from CD8^+^ and CD4^+^ TH1) and IL-17 (from γδT cell and CD4^+^ TH17), mediate endothelial dysfunction and cardiac, renal, and neural damage, aggravating hypertension [19]. Accordingly, endothelial function was found to be inversely correlated with inflammatory cytokines, TNFα, IFNγ, IL-6 and IL-17, and cytotoxic molecules, granzyme and perforin produced by CD4^+^ CD28^null^ (also CD3^+^ CD31^+^ CXCR4^+^) T-cells [48]. CD8^+^ CD28^null^ T-cells are also elevated in patients with hypertension. Youn et al. found an increased fraction of CD8^+^ CD28^null^ T-cells from a group of newly diagnosed, treatment-naïve adult patients compared with their age- and sex-matched normotensive control subjects. This population is positively correlated with the circulating levels of the CXCR3 chemoattractant, MIG (CXCL9), IP-10 (CXCL10) and I-TAC (CXCL11) [47]. CD8^+^ T-cells of hypertensive patients produce elevated levels of IFNγ, TNFα, perforin, and granzyme B. However, it is not clear whether the CD28^null^ portion possesses the same secretory profiles as the whole CD8^+^ population [47]. In children with primary hypertension, left ventricular hypertrophy (a risk factor for further CVD and morbidities) is associated with a high CD8^+^ CD28^null^ fraction [46]. Taken together, these results suggest CD8^+^ CD28^null^ T-cells are associated with the development of hypertension and CD4^+^ CD28^null^ cells engage in the pathogenic inflammation in hypertension.

Hypertension can affect both large and smell vessels. Chronic endothelial damage over time weakens the integrity of the vessel walls, increasing risk of strokes, aneurysm, renal dysfunction, and other cardiovascular complications. SARS-CoV-2 can infect endothelial cells that express ACE2, a major entry receptor for SARS-CoV-2. Patients with pre-existing, systemic endothelial vessel damage and inflammation are much more prone to severe COVID19 complications than patients who have intact vessels [75,76].

### 2.5. CVD

CVD, consisting of conditions affecting the heart and blood vessels, and comorbidities display an expanded CD4^+^ CD28^null^ T-cell population [10,20]. A pathologic increase in inflammatory cytokines, IFNγ and TNFα, and cytotoxic enzymes, granzymes A and B and perforin, contributes to deleterious cardiovascular remodeling, seen in acute coronary syndromes, plaque instability, and stroke [10,51,53]. CD4^+^ CD28^null^ T-cells from patients with acute coronary syndromes and those with at least one of atherosclerosis risk factors (hypertension, diabetes, dyslipidemia, or smoking) express higher levels of cytotoxic mediators than those with stable angina or those in a control group (although the frequencies of this population are comparable among the four groups), indicating CD4^+^ CD28^null^ cells may participate in the initial phases of atherosclerosis [51]. Circulating CD4^+^ CD28^null^ cell counts in patients with end-stage renal disease are positively correlated with increased serum levels of C-reactive protein (an inflammatory marker), impaired flow-mediated vasodilation, and increased intima-media thickness of the carotid artery. These CD4^+^ CD28^null^ cells express higher levels of pro-inflammatory and cytotoxic mediator than CD4^+^ CD28^+^ cells, strengthening their role in mediating the early development of atherosclerosis [53]. Recent studies on patients with rheumatoid arthritis (RA) and systemic lupus erythematosus echo these results: expansion of CD4^+^ CD28^null^ cells correlates with significantly higher carotid-intima media thickness and lower brachial artery flow-mediated endothelium-dependent dilation [54,77]. Moreover, CD4^+^ CD28^null^ cells are also a risk factor for poorer prognostic outcomes in CVD [57,58]. Interestingly, patients with advanced atherosclerotic disease and concurrent elevations in CD4^+^ CD28^null^ cells have a worse prognosis; however, there is an inverse relationship between high CD4^+^ CD28^null^ cells and first-time coronary events in a population-based cohort [52]. These conflicting findings warrant the need for more research, especially on the antigen specificity of these cells and related comorbidities.

CD8^+^ CD28^null^ T-cells are also associated with cardiovascular disorders. A Korean study showed that the frequency of CD8^+^ CD57^+^, CD8^+^ CD28^null^ and cytomegalovirus-specific CD8^+^ T-cells are independently correlated with arterial stiffness, a well-known predictor of future cardiovascular events, among which cytomegalovirus-specific CD8^+^ T-cells produce IFNγ and TNFα and are highly abundant in the CD8^+^ CD57^+^ fraction [49]. In another study, patients with acute coronary syndrome and stable angina accumulate in blood a population of IFNγ-producing CD8^+^ CD56^+^ T-cells that contain more CD28^null^ cells than the CD56^−^ cells, indicating a harmful nature of CD8^+^ CD28^null^ T-cells [50].

COVID-19 has been documented to cause acute myocardial infarction and ischemic strokes [78,79]. Patients who already have deleterious endothelial damage, cardiovascular remodeling, and atherosclerosis have an increased risk of experiencing more frequent and severe cardiac events from a COVID-19 infection.

### 2.6. Cancer

Malignancies are associated with immune insufficiency, especially CD8^+^ cytotoxic T (CTL) cell dysfunction, including tolerance, anergy, exhaustion, and senescence [12,80,81]. Enriched CD8^+^ CD28^null^ (or CD57^+^) senescent T-cells are found in peripheral blood and tumor microenvironment of patients with various solid and hematopoietic tumors (reviewed by [14]). Expansion of this population appears to be driven by the tumor microenvironment itself, contributing to immune compromise [62,82,83]. In a study on head and neck cancers, tumor removal causes the expanded CD8^+^ CD28^null^ cells to return to normal levels [82]. The frequency of CD8^+^ CD28^null^ T-cells in metastatic breast cancer is independently correlated with shortened survival time [61]. In melanoma, expanded CD8^+^ CD28^null^ cells express enhanced levels of NK associated receptors and perforin, impacting their effector function [63]. In addition to CD8^+^ CD28^null^ cells, CD4^+^ CD28^null^ T-cells also expand in cancer patients and are associated with poor prognosis. For example, glioblastoma patients with higher numbers of circulating CD4^+^ CD28^null^ T-cells have poor post-surgery survival [84].

CTL exhaustion has been a target for checkpoint inhibition therapy against PD1 and CTLA4 receptors and has achieved paramount efficacy in many cancer types, especially melanoma and non-small cell lung carcinoma [85,86]. However, a recent study on a small cohort of melanoma patient showed that high CD4^+^ and CD8^+^ CD28^null^ (or CD57^+^) senescent T-cells may result in resistance to checkpoint inhibitor treatment [87]. In non-small cell lung carcinoma, hyperprogressive disease is correlated with systemic expansion of CD4^+^ CD28^null^ cells after the first cycle of anti-PD-1/PD-L1 immunotherapy [64].

Malignancy is a known hypercoagulable state [88]. As COVID-19 can also cause hypercoagulability [89,90], cancer patients infected with SARS-CoV-2 could be at an increased risk of arterial and/or venous clot formation.

In summary, CD28^null^ senescent T-cells accumulate in cancer patients and CD8^+^ and CD4^+^ CD28^null^ populations may both promote disease progression. Coincident COVID-19 increases the risk of coagulopathy in cancer patients.

## 3. Mechanisms Underlying CD28^null^ Cells-Associated Adverse Consequences

COVID-19 is known to elicit intensive immune/inflammatory responses and drive the expansion/formation of CD28^null^ senescent T-cells, which together worsen prognosis of the chronic disorders (see discussion above). However, it is not well understood how expanded senescent T-cells in aging-related chronic diseases adversely impact COVID-19. To better understand the detrimental effects of these senescent T-cells, we summarize their molecular and cellular features and analyze their influence on the immune system and associated consequences.

### 3.1. Decline of Lymphocytic Diversity and Naïve/Effector Pools

**Figure 2:**
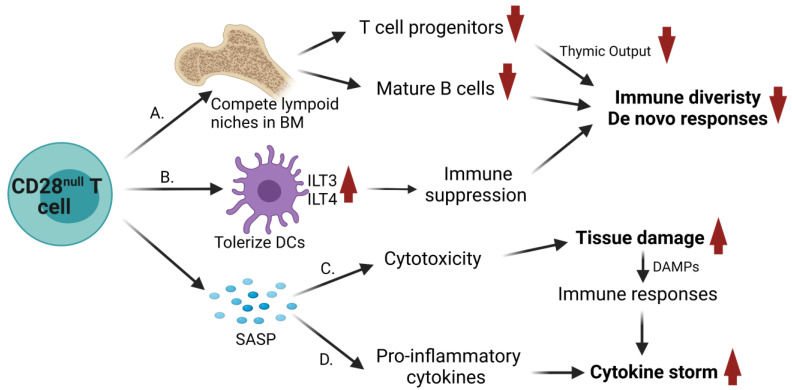
Molecular and cellular basis whereby CD28^null^ senescent T-cells lead to adverse outcomes. (A) CD28^null^ senescent T-cells resist to apoptosis and migrate to bone marrow (BM), where they compete in limited lymphoid niches, which leads to decreased output of mature B cells and T-cell progenitors. A decrease in T-cell progenitors further results in thymic dystrophy and impaired T-cell development. Decreases in B and T-cell replenishment lead to narrowed antigenic diversity. (B) CD28^null^ senescent T-cells interact with dendritic cells (DCs) and tolerize DCs by induction of high levels of inhibitory receptors, ILT3 and ILT4, and repression of CD28/CTLA4 ligands, CD80 and CD86, contributing to immune suppression. (C-D) CD28^null^ senescent T-cells possess a SASP. (C) After receiving stimuli from alternative costimulatory molecules, OX40 and 4-1BB, and NK-like receptors, CD28^null^ cells actively express cytotoxic mediators, perforin and granzymes, which mediate unrestricted tissue damage and release of damage-associated molecular patterns (DAMPs). DAMPs enhance immune responses. (D) CD28^null^ cells also produce pro-inflammatory cytokines, such as IL-6, IL-17, TNFα, and IFNγ, contributing to worsening cytokine release syndrome (“cytokine storm”) in infectious diseases, such as COVID-19.

Naïve T-cells recirculate between blood and secondary lymphoid organs by expressing CCR7 and CD62L. Upon activation and differentiation, naïve T-cells become effector T-cells that down-regulate CCR7 and CD62L and express new integrins and selectin ligands for relocation to specific peripheral tissues. Effector T-cells are eliminated by clonal contraction when the offending agent is cleared. A small portion of antigen experienced T-cells develops into long-live effector (T_EM_), central (T_CM_) or tissue-resident (T_RM_) memory cells. T_RM_ cells with specific integrins and selectin ligands localize to peripheral tissues. T_CM_ cells express CCR7 and CD62L and, similar to naïve T-cells, reside in secondary lymphoid organs. Effector and T_EM_ cells are CCR7^−^ CD62L^−^ but can localize to secondary lymphoid organs in a CXCR3 or P-selectin-dependent manner [91]. In addition to secondary lymphoid organs, bone marrow (BM) is another reservoir of memory T-cells. BM tropism of memory T-cells depends on integrin VLA-4 (α4β1) and CXCR4; the latter strongly responds to BM chemokine CXCL12 [92]. The frequency of CD4^+^ CD28^null^ T-cells is correlated with endothelial dysfunction in hypertensive patients and a cardiovascular risk in systemic lupus erythematosus [48,56]; their expression of CXCR4 suggests a BM homing property. Indeed, clonally expanded CD28^null^ T-cells are enriched in bone marrow [27,93]. The existing memory T-cells in BM compete with de novo generated memory T-cells migrating to BM [94]. Due to the limited spaces, the presence of increased CD28^null^ T-cells in BM decreases the output of mature B cells and T-cell progenitors. The latter further results in thymic dystrophy and impairment of T-cell replenishment. These together lead to a shrinkage of naïve and effector memory B and T-cell pools with narrowed diversity (Figure 2). As a consequence, accumulation of CD28^null^ T-cells creates an overall decline of immune responses in both humoral and cellular arms [10,14,27,69]. It has been shown that expansion of CD8^+^ CD28^null^ T-cells predicts poorer antibody responses to influenza vaccination in the elderly [95]. For COVID-19, expansion of CD28^null^ T-cells results in poor immune responses, including neutralizing antibody and anti-viral CTL response, which could lead to worsened outcomes.

### 3.2. Immune Suppression

CD8^+^ CD28^null^ cells tolerize dendritic cells (DCs) through induction of high levels of inhibitory receptors, ILT3 and ILT4, and repression of CD28/CTLA4 ligands, CD80 and CD86 [96,97]. The tolerogenic DCs anergize CD4^+^ T-cells [97] and promote CD4^+^ T-cells regulatory activity [96] (Figure 2). Tumor-associated monocytic myeloid-derived suppressor cells (MDSCs) possess similar features, such as hyper-expression of ILT3 and ILT4 [98,99], and can educate CD4^+^ Foxp3^−^ IL-10^+^ regulatory T (T_R_) cells [100]. In addition, MDSCs may participate in immunosenescence induction [101]. It is not clear whether CD4^+^ CD28^null^ cells can also tolerize DCs, although they have similar cytotoxic and pro-inflammatory characteristics as their CD8^+^ counterparts.

In addition to repeated antigen stimuli, naturally occurring CD4^+^ CD25^hi^ Foxp3^+^ T_R_ cells and tumor-associated regulatory γδT-cells have been shown to induce a senescent phenotype on naïve and responder T-cells (Figure 1), characterized by down-regulation of CD27 and CD28 and expression of senescence-associated beta-galactosidase (SA-β-gal) [102,103]. This process is likely granzymes-dependent, because granzyme A has been shown to cause DNA damage [104], and T_R_ cells produce granzyme [105]. T_R_ cell-induced CD4^+^ and CD8^+^ CD28^null^ senescent T cells are potent suppressor. Their function is dependent on DNA damage-associated p38 and ERK1/2 cascades [102,106]. A portion of CD8^+^ CD28^null^ cells from patients with glioblastoma express Foxp3 and are associated with a tolerogenic phenotype of tumor-infiltrating APCs that express ILT2, ILT3, and ILT4 [107]. Whether CD8^+^ CD28^null^ Foxp3^+^ T_R_ cells behavior as natural T_R_ cells and reinforce immunosenescence needs to be studied. Senescent T-cells-mediated immune suppression may contribute to immune insufficiency. In COVID-19, severe illness is largely attributed to lack of viral control due to immune insufficiency, such as under-expression of IFN-I.

### 3.3. Direct Cytotoxicity

With down-regulation of CD28, both CD4^+^ and CD8^+^ CD28^null^ T-cells gain expression of NK cell activating receptors, including CD94/NKG2 heterodimers, NKG2D/NKG2D homodimer and KIR2DL4, and produce cytotoxic mediators, granzymes and perforin [32,107,108]. Despite their down-regulation of CD28, CD4^+^ CD28^null^ cells express high levels of TNFR family costimulatory receptors OX40 and 4-1BB, which mediate their cytotoxic function [109]. Stimulation of OX40 and 4-1BB leads to release of perforin and granzyme B, contributing to the SASP of these senescent T-cells. In addition, signals from the NK-like costimulatory receptor NKG2D in CD8^+^ T-cells cause cytotoxic activation in a TCR-dependent or -independent manner [12,110,111]. Tissue damage caused by the cytotoxicity of CD28^null^ T-cells induces damage-associated molecular patterns (DAMPs), contributing to inflammatory responses (Figure 2). DAMPs, such as HMGB1, S100A8/A9 and SP-A, are elevated in COVID-19 patients compared to healthy subjects [112]. SP-A levels positively correlate with the amounts of inflammatory cytokines and negatively correlate with time elapsed since symptom onset [112]. HMGB1 promotes inflammatory neutrophil extracellular traps and is suggested as a therapeutic targets in severe COVID-19 [113,114]. In summary, appropriate triggers can elicit cytotoxicity, especially antigen-independent cytotoxicity, of CD28^null^ T-cells and lead to an increased risk of unrestrained tissue damage in the aging-related chronic diseases and COVID-19.

### 3.4. Contribution to Cytokine Release Syndrome

Cytokine release syndrome (or cytokine storm) is involved in many inflammatory processes, such as infections, autoimmune diseases, and acute graft-versus-host disease. Mediators of cytokine storms include cytokines (such as IL-6, IL-1β, and TNFα), chemokines, and tissue factors. Cytokines, IL-6, IL-1β, and TNFα are essential in the systemic inflammation due to their ability in amplifying innate and adaptive immune responses [115,116] (Figure 2). In COVID-19, cytokine storms feature profoundly high levels of IL-6 and are associated with higher mortality [116,117,118,119,120,121].

As a part of SASP, CD28^null^ T-cells produce increased amounts of pro-inflammatory cytokines, IL-6, IL-17, TNFα, and IFNγ (see details in Table 1) after receiving appropriate stimuli from TCR ligation, alternative costimulation of OX40 and 4-1BB and activating NK-like receptors. Among these, IFNγ drives activation of monocytes and converts them into M1 macrophages, which produce massive pro-inflammatory cytokines, including IL-6, IL-1β, and TNFα. IL-17 also has many downstream effects on pro-inflammatory cascades, including induction of cytokines [G-CSF (responsible for granulopoiesis and recruitment of neutrophils), IL-6, IL-1β, and TNFα], chemokines, and matrix metalloproteinases (contributing to tissue remodeling and damage). Myeloid (both monocytic and neutrophilic) hyper-responsiveness is a common phenomenon of severe COVID-19 [1,2,3]. Taken together, the secretory mediators produced by CD28^null^ T-cells along with enhanced myeloid responses reinforce systemic inflammation, leading to deleterious outcomes in COVID-19.

## 4. Potential Treatments

As discussed earlier, evidence suggests that CD28^null^ T-cells lead to serious negative consequences in patients with chronic diseases and COVID-19. Targeting these cells may prove beneficial. The therapeutic strategies below are focused on removal of these senescent cells and restoration of functional naïve/effector T-cell pools.

### 4.1. Re-sensitization to Apoptosis

Because CD28^null^ senescent T-cells are functionally abnormal and overwhelm limited lymphoid spaces, one therapeutic strategy is to remove the population. Both CD8^+^ CD28^null^ and CD4^+^ CD28^null^ T-cells have mechanisms to evade apoptosis. The extrinsic pathway of apoptosis is triggered by ligation of death receptors, such as Fas (CD95). CD28^null^ T-cells are resistant to Fas-mediated apoptosis (FasL) [16,18,122]. The apoptotic resistance of CD28^null^ T-cells relies on their down-regulation of pro-apoptotic molecules, Fas, Bim, and Bax [122], or up-regulation of anti-apoptotic molecule Bcl2 [18]. CD4^+^ CD28^null^ T-cells display hyperactive ERK1/2, resulting in Bim phosphorylation and proteasomal degradation [122]. Treatment in vitro with proteosome inhibitor MG-132 preserves phosphor-Bim and restores apoptotic sensitivity in CD4^+^ CD28^null^ cells.

Statins, a drug class broadly used to lower cholesterol, appears to have immunologic impacts beyond their traditional lipid-lowering mechanisms. Statins have been shown to slightly decrease the percentage of CD4^+^ CD28^null^ T-cells in patients with unstable angina [123]. In acute coronary syndromes, rosuvastatin treatment dramatically decreases CD4^+^ CD28^null^ T-cells [124]. Rosuvastatin induces apoptosis in CD4^+^ CD28^null^ T-cells via down-regulation of Bcl2 [124]. Interestingly, atorvastatin and rosuvastatin do not induce significant apoptosis of these cells in vitro [122], suggesting that statins may indirectly act on T-cells. Because statins induce pro-inflammatory cytokine IL-18 and may contribute to cytokine storms [125], the side effects of statins are concerning for COVID-19 patients [126,127]. Nevertheless, clinical observations plausibly demonstrate that administration of statins before or after COVID-19 diagnosis is associated with a lower risk of developing severe disease, a faster time to recovery, and a lower mortality rate [128,129,130].

Steroids are well-known to induce apoptosis in lymphocytes and suppress their function [131]. CD8^+^ CD28^null^ senescent T-cells from COPD patients are resistant to steroids due to decreased expression of glucocorticoid receptor [74]. This population also expresses Pgp1 [74], a major drug efflux pump responsible for multidrug resistance in cancer. In the presence of very low-dose of cyclosporine A (a Pgp1 inhibitor), corticosteroid treatment results in inhibition of pro-inflammatory cytokines in CD8^+^Pgp1^+^CD28^null^ NKT-like cells [22,132]. CD28^null^ T-cells from COPD patients express a low level of histone deacetylase SIRT1, which is associated with their pro-inflammatory phenotype [42]. In the presence of SIRT1 activators, such as theophylline, curcumin or resveratrol, treatment with prednisolone increases SIRT1 expression and restores steroid sensitivity, which in turn inhibits pro-inflammatory cytokine secretion from these cells [42]. Although above studies have shown either inhibition of Pgp1 or activation of SIRT1 can restore steroid sensitivity in CD28^null^ T-cells, further investigation is required to determine whether these treatments can re-sensitize these cells to apoptosis in clinical settings.

Senolytics, a set of naturally occurring or synthetic compounds that selectively clear senescent cells, is attracting broad interests for treating aging- and chronic diseases-associated senescence [133,134]. Senolytics have shown efficacy in early clinical trials for idiopathic pulmonary fibrosis and diabetic chronic kidney disease [135,136]. In vitro, the combination of dasatinib (a tyrosine kinase inhibitor) and quercetin (a naturally occurring flavonoid) causes apoptosis of both senescent human primary adipocyte progenitor cells and senescent umbilical cord vein endothelial cells (HUVECs), but not their nonsenescent counterparts [137]. A murine study demonstrates that treatment with the senolytic cocktail, dasatinib plus quercetin, decreases naturally occurring senescent cells. Additionally, the treatment alleviates physical dysfunction in both senescent cell-transplanted young mice and naturally aged mice, bolstering post-treatment survival [138]. Senolytics-mediated clearance of senescent cells occurs via modulation of apoptotic factors, such as ephrins and Bcl2 family members [133]. Since senolytics are not specific for CD28^null^ senescent T-cells, their drug effects may act directly on these cells or through clearing other senescent cells. Several clinical trials are investigating potential benefit of senolytics on senescence-associated severe COVID-19 [139].

### 4.2. Targeting the Costimulatory Pathways

Loss of costimulatory receptor CD28 in T-cells leads to metabolic and epigenetic alterations, rendering the cells senescent. It has been shown that forced expression of CD28 in CD8^+^ CD28^null^ CMV- and HIV-specific CD8^+^ T-cells reconstitutes their ability to produce IL-2, which sustains an autocrine proliferative response after antigen recognition [140]. After IL-12 exposure, CD4^+^ CD28^null^ senescent T-cells re-express CD28 and gain CD25 and CD40 ligands, suggesting that IL-12, at least in part, functionally rescues senescent CD4^+^ T-cells [141]. Another potential treatment option is inhibiting TNFα, which down-regulates CD28 expression on T-cells [142]. In some studies, TNFα blockade decreases the frequencies of CD28^null^ senescent T-cells in patients with RA and unstable angina [143,144]; however, other studies did not observe this effect of TNFα [13,145]. Whether restoration of CD28 can re-sensitize CD28^null^ senescent T-cells to apoptosis is to be investigated.

Abatacept, a CTLA-4Ig fusion protein, functions by binding to B7 ligands CD80/CD86 and blocking their interaction with CD28 on T-cells. Abatacept decreases circulating CD4^+^ and CD8^+^ CD28^null^ T-cells in a 48-week clinical trial for RA, and shows clinical improvement of symptoms [146]. In another study, RA patients receiving abatacept for >5 years have comparable numbers and frequencies of CD4^+^ CD28^null^ T-cells compared to healthy controls, correlating with decreased disease activity [147]. These results suggest that attenuated stimulation of CD28 on effector cells decreases de novo generation of CD28^null^ cells.

CD4^+^ CD28^null^ cells express high levels of OX40 and 4-1BB during activation. Stimulation of OX40 and 4-1BB leads to hyper-secretion of pro-inflammatory cytokines and cytotoxic molecules [109]. Targeting the alternative costimulatory receptors may lessen the cytotoxic and pro-inflammatory function of CD4^+^ CD28^null^ cells and benefit COVID-19 patients.

### 4.3. Targeting the Maintenance of Senescent Cells

IL-15 and IL-6 are highly expressed in BM and promote the development and maintenance of CD28^null^ T-cells [29,148]. Due to DNA damage repair pathways being compromised, CD8^+^ CD28^null^ cells have increased apoptosis compared to CD8^+^ CD28^+^ cells when exposed to etoposide, a chemotherapeutic topoisomerase II inhibitor [149]. Administration of IL-15 prevents etoposide-induced apoptosis of CD8^+^ CD28^null^ cells, suggesting a role of IL-15 in the survival of CD28^null^ senescent cells. Another example of deleterious effects of IL-15 can be seen in multiple sclerosis (MS). In MS, IL-15 is mainly produced by astrocytes and infiltrating macrophages in inflammatory lesions and selectively attracts CD4^+^ CD28^null^ T-cells via induction of chemokine receptors and adhesion molecules [70]. In addition, IL-15 increases proliferation of CD4^+^ CD28^null^ cells and their production of GM-CSF, cytotoxic molecules (NKG2D, perforin, and granzyme B), and degranulation capacity. In BM, levels of ROS are positively correlated with the levels of IL-15 and IL-6. When incubated with ROS scavengers, vitamin C and N-acetylcysteine (NAC), BM mononuclear cells express decreased amounts of IL-15 and IL-6 [29], which may ultimately decrease CD28^null^ cells and therefore, allow other immune cell populations to re-establish in BM. In murine studies, vitamin C and NAC improve generation and maintenance of memory T-cells in the elderly [150]. In a small cohort phase I trial, methylene blue-vitamin C-NAC treatment appears to increase the survival rate of COVID-19 patients admitted to intensive care [151], which targets oxidative stress and may improve BM function via restriction of senescent cells.

### 4.4. Preventing Senescence

CD4^+^ Foxp3+ T_R_ cells have been shown to drive CD4^+^ and CD8^+^ T-cells to down-regulate CD28 and gain a senescent phenotype with suppressive function. T_R_ cells activate ataxia-telangiectasia mutated protein (ATM), a nuclear kinase that responds to DNA damage. Activated ATM then triggers MAPK ERK1/2 and p38 signaling that cooperates with transcription factors STAT1/STAT3 to control responder T-cell senescence [106,152]. Pharmaceutical inhibition of ERK1/2, p38, STAT1, and STAT3 pathways in responder T-cells can prevent T_R_-mediated T-cell senescence. TLR8 agonist treatment in T_R_ and tumor cells inhibits their ability to induce senescent T-cells [83,102]. In tumor microenvironment, cAMP produced by tumor cells is directly transferred from tumor cells into target T-cells through gap junctions, inducing PKA-LCK inhibitory signaling and subsequent T-cell senescence, whereas TLR8 signals down-regulate cAMP to prevent T-cell senescence [83]. In addition, CD4^+^ CD27^−^ CD28^null^ T-cells have abundant ROS [152], which induces DNA damage [153] and activates metabolic regulator AMPK [154]. AMPK recruits p38 to the scaffold protein TAB1, which causes autophosphorylation of p38. Signaling via this pathway inhibits telomerase activity, T-cell proliferation, and the expression of key components of the TCR signalosome, resulting T-cell senescence [152]. Autophagy is well-known for intracellular homeostasis by removal of damaged organelles and intracellular waste. However, in the presence of intensive mitochondrial ROS production, sustained p38α activation leads to phosphorylation of ULK1 kinase. This triggers massive autophagosome formation and basal autophagic flux, resulting in senescence instead of apoptosis of cancer cells [155]. In nonsenescent T-cells, activation of p38 by a specific AMPK agonist reproduces senescent characteristics, whereas silencing of AMPKα (a subunit of AMPK) or TAB1 restores telomerase and proliferation in senescent T-cells [152]. Therefore, blockade of p38 and relevant pathways can prevent T-cell senescence and is promising to restore the function of senescent cells, which could have far-reaching therapeutic effects on COVID-19 and age-related diseases.

In addition, early detection and prompt treatment of chronic diseases may prevent or diminish the accumulation of CD28^null^ senescent T-cells and decrease the risk of developing comorbidities and severe infections.

## 5. Conclusions

An elevated proportion of CD28^null^ T-cells occurs in aging and chronic conditions, contributing to disease development and pathogenic inflammation. The clinical management of CD28^null^ cells is challenging because they create a paradoxical pro-inflammatory, cytotoxic environment while also instigating suppression to protective immune responses. Immunotherapy options we currently have include, but are not limited to re-sensitization to apoptosis using statins, steroids, and senolytics, and prevention of de novo generation by targeting costimulatory pathways, DNA damage-associated ATM-p38 pathway, and nutrient status regulated AMPK-p38 pathway. Although aging is unavoidable, cell senescence can be modulated. Improved preventive care and management of chronic diseases may decrease the rate of inflammaging, senescence, and accumulation of CD28^null^ T-cell populations. Addressing the pathology caused by senescent T-cells would not only improve quality of life of patients with aging-related chronic diseases, but also aid in reducing morbidity and mortality of patients who also suffer from COVID-19.

## Figures and Tables

**Table 1:** CD28^null^ senescent T-cells in aging and underlying conditions.

Factors	CD28^null^ Subset	Adverse Effects
Aging	CD8^+^	↓Naïve T-cell pool → ↓Antigenic diversity → ↓Immune response [10,14] ↓B cell Population [27] ↑T-cell senescence, inferred [12] ↑Progression of Alzheimer’s disease [28] ↑IL-6; ↑IFN-γ → ↑IL-15 [29] ↑CD94/NKG2 → ↑Cytotoxicity [10,14] ↓Autophagy [30]
	CD4^+^	↑NK receptors → ↑Inflammation and cytotoxicity [10,13,31] ↓Dnmt1and Dnmt3a → ↑KIR, perforin, and CD70 [32] ↑CX3CR1 [33]
Diabetes	CD8^+^	↓Immune response in children [34] ↓Immune response in adults [35] ↑Risk of developing hyperglycemia [36] ↑ROS; ↑Glycolysis → ↑T2D development [37]
	CD4^+^	↑Risk of acute coronary syndrome [13] ↑IL-17 in CD4^+^ CD28^null^ NKG2D^+^ T cells → ↑Systemic inflammation; ↑HbA1c [38] ↑HbA1c and urinary albumin creatinine ratio [39] ↑Risk of cardiovascular events [21]
COPD	CD8^+^	↑Pro-inflammatory cytokines, granzyme and perforin [40] ↓Glucocorticoid receptor → ↑Steroid resistance [22] ↓ Glucocorticoid receptor and Hsp90 → ↑IFNγ [41] ↓SIRT1 → ↑IFNγ, TNFα, steroid resistance, and disease severity [42] ↓Apoptosis; ↑Potential of tissue injury [43]
	CD4^+^	↑IFNγ → matrix metalloproteinases and tissue destruction; ↑Apoptosis of lung epithelium [44] ↑Natural killer-like T-cell receptors CD94 and CD158; ↑Intracellular perforin and granzyme B; ↑TNF increase and IFNγ only increase at early disease [45]
Hypertension	CD8^+^	↑Development of left ventricular hypertrophy (LVH) [46] ↑Granzyme B and Perforin; ↑CXCR3 chemokines, MIG, IP-10 and I-TAC; ↑IFNγ and TNFα [47]
	CD4^+^	↑IFNγ, IL-6, IL-17, and TNFα; ↑Granzyme, and perforin; ↓Flow-mediated dilation [48]
CVD	CD8^+^	↑IFNγ and TNFα [49] ↑IFNγ [50]
	CD4^+^	↑Risk of plaque instability, acute coronary syndromes, and stroke [10] ↑IFNγ and TNFα; ↑Perforin, granzyme A, and granzyme B [51] ↑Cytotoxins and cytokines [20] ↑Risk of complications in follow-up surgeries; ↓Risk of first-time coronary event [52] End-stage renal disease: ↑IFNγ; ↓IL-4; ↑Granzyme B and perforin; ↓Flow-mediated vasodilation; ↑Carotid-intima media thickness (cIMT) [53] Pediatric T1D: ↑Aortic stiffness, and cIMT [39] Rheumatoid arthritis: ↑cIMT; ↓FMEDD [54] Kidney transplantation: ↑Risk of an atherosclerotic vascular event [55] Systemic lupus erythematosus: ↑Anti-dsDNA and anti-SSA/Ro; ↑ TNFα, IL-8, IFNα, and B lymphocyte stimulator [56] Acute coronary syndrome: ↑Non-ST-segment elevation ACS (NSTEACS) versus ST-segment elevation myocardial infarction (STEMI) [57] Chronic heart failure: ↑Mortality rate [58]
Cancer	CD8^+^	Non-small lung cancer: ↑Poor prognosis [59] Lung cancer: ↑Foxp3 mRNA in CD8^+^ CD28^null^ T-cells; ↓Immune response, inferred [60] Metastatic breast cancer: ↑IL-6 and IL-10; ↓Progression-free survival [61] Colorectal cancer: ↑Suppression of cytotoxic function of T-cells; ↑Suppression of T-cell proliferation [62] Melanoma: ↑NK receptors, CD94, NKG2A, CD56, CD57, CD16, and CD244; ↑Perforin [63]
	CD4^+^	Non-small lung cancer (with immunotherapy): ↑Risk of hyperprogressive disease after anti-PD-1/PD-L1 immunotherapy [64] Cervical cancer: ↑NKG2D; ↑Perforin [65]

↑, up-regulation. ↓, down-regulation. →, leading to.
